# Access to Essential Medications for Burn Management Among Rural Communities in India: A Community-Based Cross-Sectional Study

**DOI:** 10.7759/cureus.116927

**Published:** 2026-09-25

**Authors:** Vidya Kiran Bammidi, Ansha Shrimal, K Murali, Dishari Das, J. Dhanuja Rani, Parth Suthar, Rahul VC Tiwari

**Affiliations:** 1 Department of General Surgery, Gayatri Vidya Parishad (GVP) Medical College, Visakhapatnam, IND; 2 Department of Pediatric and Preventive Dentistry, Bhabha College of Dental Sciences, Bhopal, IND; 3 Department of Plastic Surgery, Kilpauk Medical College and Hospital, Chennai, IND; 4 Department of Conservative Dentistry and Endodontics, Kalinga Institute of Dental Sciences, Kalinga Institute of Industrial Technology (KIIT) (Deemed to be University), Bhubaneswar, IND; 5 Department of Oral and Maxillofacial Pathology, Government Dental College and Research Institute, Ballari, IND; 6 Department of Oral and Maxillofacial Surgery, Narsinhbhai Patel Dental College and Hospital, Visnagar, IND

**Keywords:** access to medicines, affordability, burn management, cross-sectional survey, essential medications, india, rural health

## Abstract

Introduction

Burn injuries remain a major public health concern, particularly in rural communities of low- and middle-income countries, where timely access to essential medications is often limited by socioeconomic and healthcare-related barriers. This study assessed access to essential burn management medications among adults residing in rural communities and identified demographic factors associated with medication access.

Materials and methods

A community-based cross-sectional survey was conducted among rural adults over a six-month period from May to October 2023 by the Department of Oral and Maxillofacial Surgery. A multistage cluster sampling technique was used to recruit the participants. Data were collected using a study-developed, interviewer-administered questionnaire comprising 20 items, including five demographic items and 15 questionnaire items assessing five domains of medication access: accessibility, affordability, availability, utilization, and awareness. Responses were recorded using a five-point Likert scale, generating total scores ranging from 0 to 60. Descriptive statistics, independent t-tests, one-way analysis of variance (ANOVA), correlation analysis, and multiple linear regression analysis were performed, with statistical significance set at p < 0.05.

Results

Of the 600 eligible participants, 565 (94.2%) completed the survey. The mean overall access score was 39.6 ± 10.8, indicating a moderate level of access to essential burn management medications. Accessibility demonstrated the highest domain score, whereas affordability was the lowest-scoring access domain, indicating that financial affordability may represent an important challenge to medication access in the study population. Significant associations were observed between access scores and age, educational status, occupation, and monthly family income (all p < 0.001), whereas sex was not significantly associated (p = 0.094). Multiple linear regression analysis identified higher educational attainment and household income as the strongest independent predictors of better medication access, whereas increasing age independently predicted poorer access. The questionnaire demonstrated excellent internal consistency (Cronbach's α = 0.88) and good test-retest reliability (intraclass correlation coefficient {ICC} = 0.84).

Conclusions

Access to essential burn management medications among rural adults was moderate, with affordability representing the greatest challenge despite relatively good geographical accessibility. Socioeconomic status, educational attainment, occupation, and age were significantly associated with medication access. Strengthening financial protection measures, ensuring the uninterrupted availability of essential burn medications, and improving community awareness may improve equitable access to essential burn medications in rural populations.

## Introduction

Burn injuries remain a significant global public health challenge, causing substantial morbidity, disability, and mortality despite declining age-standardized rates. Recent Global Burden of Disease estimates indicate that the absolute number of burn cases is projected to increase markedly by 2050 owing to population growth and aging, particularly in low- and middle-income countries [1]. The burden of burn injuries is disproportionately higher in rural populations because of unsafe cooking practices, occupational hazards, delayed access to healthcare facilities, and limited emergency services [2]. Although advances in burn care have significantly improved survival, successful treatment depends not only on timely medical intervention but also on uninterrupted access to essential medications required for wound care, pain management, infection prevention, and supportive therapies [3]. Therefore, ensuring the availability and affordability of these medicines is fundamental to improving burn-related outcomes.

Access to essential medicines is recognized by the World Health Organization (WHO) as a key component of universal health coverage. Effective access encompasses multiple dimensions, including the availability of medicines at healthcare facilities, affordability for patients, geographical accessibility, appropriate utilization, and public awareness of their use [4]. Deficiencies in any of these domains can result in delayed treatment, poor adherence to prescribed therapy, preventable complications, prolonged recovery, and increased healthcare expenditure [5]. Rural communities often face unique challenges, such as inadequate healthcare infrastructure, irregular medicine supply, financial constraints, and long travel distances to health facilities, making equitable access to burn medications particularly difficult [6].

India has implemented several national health initiatives aimed at improving the availability of essential medicines through public healthcare facilities [6]. Despite these efforts, disparities continue to exist between urban and rural areas, and evidence regarding community-level access to medications specifically used for burn management remains limited. Most published studies have focused on the clinical management, epidemiology, or hospital-based outcomes of burn injuries, whereas comparatively little attention has been given to understanding the barriers experienced by rural populations in obtaining essential burn medications [7]. Identifying these barriers is essential for strengthening primary healthcare services, optimizing medicine supply systems, and informing policy decisions that promote equitable access to burn care.

Burn injuries vary considerably in severity and may range from minor superficial injuries that can initially be managed with appropriate first aid to deeper, extensive, or anatomically critical burns requiring prompt medical assessment and hospital-based care. Factors such as burn depth and extent; the involvement of the face, hands, feet, genitalia, major joints, or airway; chemical or electrical mechanism; and associated trauma influence the need for referral [1-3]. The present study did not assess burn severity or treatment setting; this clinical classification was therefore used only as contextual information and was not incorporated into the access score.

The study aimed to assess access to essential burn management medications among adults in rural Indian communities. Specifically, this study sought to evaluate the five key dimensions of access: accessibility, affordability, availability, utilization, and awareness, using a structured questionnaire. Furthermore, it aimed to determine the overall level of access to essential burn medications and examine the association between access and selected demographic characteristics, including age, sex, educational status, occupation, and monthly family income. This study also sought to identify independent demographic predictors of access to essential burn management medications, thereby providing evidence to support interventions and policy initiatives aimed at improving equitable access to burn care in rural populations.

## Materials and methods

This community-based cross-sectional survey was conducted by the Department of Oral and Maxillofacial Surgery at Narsinhbhai Patel Dental College and Hospital, Visnagar, Gujarat, India. The survey was conducted over a period of six months (May 2023 to October 2023) in selected rural villages served by primary healthcare facilities in and around Visnagar. The study was designed and reported in accordance with the Strengthening the Reporting of Observational Studies in Epidemiology (STROBE) guidelines for cross-sectional studies [8].

Ethical approval was obtained from the Institutional Ethics Committee of Narsinhbhai Patel Dental College and Hospital prior to the commencement of the study (approval number: NPDCH/IEC/2023/112). Participation was voluntary, and written informed consent was obtained from all respondents before their enrollment. Participant anonymity and confidentiality were maintained throughout the study by assigning unique identification numbers and securely storing all the study records.

The study population consisted of adults aged ≥18 years who had been residing in the selected rural villages for at least one year. Adults were selected to obtain information on medication access and its relationship with demographic and socioeconomic characteristics, while the minimum one-year residence criterion was applied to ensure familiarity with the local healthcare and medication-access environment and to exclude temporary residents. Individuals were included irrespective of sex, occupation, educational status, or socioeconomic background to obtain a representative assessment of community access to burn medications. Healthcare professionals, pharmacists, temporary residents, individuals with cognitive impairment or severe illness preventing participation, and those unwilling to provide informed consent were excluded. Households were eligible if they contained at least one adult aged ≥18 years who had resided in the selected rural village for at least one year and met the study's inclusion criteria. Only one eligible adult was selected from each household to avoid multiple responses from the same household and minimize within-household clustering.

In this study, the term "essential burn management medications" refers broadly to medications commonly used in the management of burn injuries, rather than to a predefined inventory of individual drugs. The questionnaire assessed participants' perceived access to such medications across five domains: accessibility, affordability, availability, utilization, and awareness. The study did not objectively verify medicine stocks or assess individual medication prescriptions.

The required sample size was estimated using Cochran's formula for cross-sectional studies:

$n=\frac{Z^{2}P(1-P)}{d^{2}}$

where Z = 1.96 for a 95% confidence level, P = 0.50, and d = 0.05. The minimum calculated sample size was 384. After applying a design effect of 1.3 for multistage cluster sampling, the sample size increased to approximately 500. To compensate for possible nonresponse and incomplete questionnaires, an additional 20% was added, resulting in a target sample size of 600. A multistage cluster sampling technique was employed, wherein rural administrative blocks were initially identified, villages were selected through simple random sampling, households were chosen using systematic random sampling, and one eligible participant from each household was selected using the Kish method [9].

A self-developed, structured questionnaire was developed by the investigators based on the study objectives, relevant literature on burn care and access to medicines, and the World Health Organization framework for access to essential medicines. The questionnaire was not adopted from a previously validated instrument. The questionnaire was originally prepared in English and subsequently translated into the local language using forward and back translations by bilingual experts to ensure semantic equivalence. Minor modifications in wording were made following expert recommendations to improve clarity and cultural appropriateness while maintaining the original meaning of each item.

The final questionnaire consisted of 20 items divided into two sections (see Appendix). Part 1 included five demographic questions regarding age, sex, educational status, occupation, and monthly/annual family income. Part 2 consisted of 15 closed-ended items evaluating access to essential medications for burn management across five domains: accessibility, affordability, availability, utilization, and awareness, with three items allocated to each domain. Each statement was answered using a five-point Likert scale, where Never = 0, Rarely = 1, Sometimes = 2, Often = 3, and Always = 4. One negatively worded item related to financial barriers to treatment was reverse-scored. Consequently, the total questionnaire score ranged from 0 to 60, with higher scores indicating better access to essential medications. Scores of 0-15, 16-30, 31-45, and 46-60 represented very poor, poor, moderate, and good access to burn medications, respectively. These categories were developed for descriptive purposes and were not based on externally validated clinical thresholds.

To establish content validity, the questionnaire was evaluated by a multidisciplinary panel of seven experts, including specialists in community medicine, oral and maxillofacial surgery, burn surgery, plastic surgery, general surgery, public health, and epidemiology. The experts independently assessed each questionnaire item for relevance, clarity, simplicity, and necessity, using a four-point rating scale. The Item Content Validity Index (I-CVI) and Scale Content Validity Index (S-CVI) were calculated, with acceptable thresholds of ≥0.78 and ≥0.90, respectively. The recommendations provided by the experts were incorporated before finalizing the questionnaire for field use.

Prior to the main survey, a pilot study involving 30 adults from a rural community not included in the final sample was conducted to evaluate the questionnaire's comprehensibility, feasibility, language clarity, and average administration time. Responses obtained during the pilot testing were excluded from the final analysis. The internal consistency reliability of the finalized questionnaire was assessed using Cronbach's alpha coefficient, with a value of 0.88, indicating excellent internal consistency. The reliability of the individual domains ranged from 0.76 to 0.82, confirming acceptable consistency across all questionnaire sections. In addition, test-retest reliability was evaluated by readministering the questionnaire to a subset of 50 participants after a two-week interval, yielding an intraclass correlation coefficient (ICC) of 0.84, demonstrating good reproducibility of the instrument.

Data were collected by trained investigators from the Department of Oral and Maxillofacial Surgery, who were proficient in the local language. Before the commencement of the survey, all investigators underwent standardized training regarding interviewing techniques, ethical considerations, questionnaire administration, and quality assurance procedures to ensure uniformity during data collection. House-to-house visits were conducted during the daytime, and eligible participants were individually interviewed in a private setting after obtaining written informed consent. Each interview required approximately 15-20 minutes for completion. To maximize participation, investigators made up to three separate visits to households where eligible respondents were unavailable during the initial visit to the household. Households that could not be contacted after three attempts or individuals who declined to participate were considered non-responders and were replaced by the next eligible household identified through the systematic sampling procedure. Completed questionnaires were reviewed daily by the field supervisor to identify missing responses or inconsistencies prior to data entry.

All completed questionnaires were coded using unique identification numbers and entered independently into Microsoft Excel (Microsoft Corp., Redmond, WA) by two trained data-entry operators. Double data entry and cross-verification were performed to minimize transcription errors, and discrepancies were resolved by comparing the entries with the original questionnaires. The cleaned dataset was imported into IBM SPSS Statistics version 27.0 (IBM Corp., Armonk, NY) for statistical analysis. Descriptive statistics were calculated for all the demographic variables and questionnaire responses. Continuous variables were expressed as mean ± standard deviation (SD) or median with interquartile range (IQR), depending on the data distribution, whereas categorical variables were presented as frequencies and percentages. The normality of continuous variables was assessed using the Shapiro-Wilk test. The internal consistency of the questionnaire was evaluated using Cronbach's alpha coefficient. Associations between access scores and demographic variables were analyzed using independent samples t-test or one-way analysis of variance (ANOVA) for normally distributed variables. Correlations between continuous variables were assessed using Pearson's or Spearman's correlation coefficients, as appropriate. Finally, multiple linear regression analysis was performed to identify the independent predictors of access to essential medications after adjusting for potential confounding variables. Statistical significance was set at p < 0.05.

## Results

A total of 600 eligible adults from rural communities were approached to participate in the study. After excluding incomplete questionnaires and non-responders, 565 participants (94.2%) were included in the final analysis of the study.

Participant characteristics

Participants aged 31-45 years constituted the largest age group, followed by those aged 18-30 years, whereas participants older than 60 years accounted for 90 (15.9%) participants. Women slightly outnumbered men in the study. Nearly one-third of the participants had no formal education, while graduates represented 55 (9.7%) of the participants. Most participants were daily wage workers or farmers. Most households reported a monthly family income of less than ₹20,000, reflecting the predominantly rural socioeconomic profile of the study population (Table 1).

**Table 1:** Demographic characteristics of the study participants (N = 565). Data are presented as n (%). N: total number of participants

Characteristic	Category	n (%)
Age (years)	18-30	152 (26.9%)
	31-45	189 (33.5%)
	46-60	134 (23.7%)
	>60	90 (15.9%)
Sex	Male	276 (48.8%)
	Female	289 (51.2%)
Educational status	No formal education	168 (29.7%)
	Primary school	145 (25.7%)
	Secondary school	119 (21.1%)
	Higher secondary	78 (13.8%)
	Graduate and above	55 (9.7%)
Occupation	Farmer	136 (24.1%)
	Daily wage worker	147 (26%)
	Homemaker	118 (20.9%)
	Government/private employee	68 (12%)
	Business	42 (7.4%)
	Student	27 (4.8%)
	Unemployed	27 (4.8%)
Monthly family income	<₹10,000	186 (32.9%)
	₹10,001-20,000	156 (27.6%)
	₹20,001-40,000	121 (21.4%)
	₹40,001-60,000	59 (10.4%)
	>₹60,000	43 (7.6%)

Association between demographic characteristics and access to essential medications

Significant differences in access scores were observed across age groups (p < 0.001), educational status (p < 0.001), occupation (p < 0.001), and monthly family income (p < 0.001). Participants aged 18-30 years demonstrated the highest mean access score (38.4 ± 8.2), whereas those aged >60 years had the lowest (31.8 ± 9.5). Access scores increased progressively with educational attainment, from 31.2 ± 8.4 among participants with no formal education to 43.8 ± 7.9 among graduates. Similarly, participants with a monthly family income of >₹60,000 reported significantly higher access scores (46.5 ± 7.3) than those earning <₹10,000 (30.8 ± 8.2). No statistically significant association between sex and access score was identified in this study (p = 0.094) (Table 2).

**Table 2:** Association between demographic characteristics and access to essential medications for burn management. Data are presented as mean ± standard deviation (SD). An independent samples t-test was used for sex, and one-way analysis of variance (ANOVA) was used for age, educational status, occupation, and monthly family income. Effect size was expressed as Cohen's d for the t-test and eta squared (η²) for ANOVA. *A two-tailed p-value of <0.05 was considered statistically significant. df: degrees of freedom

Characteristic	Category	Access score, mean ± SD	Test statistic (df)	P-value	Effect size
Age (years)	18-30	38.4 ± 8.2	F (3,561) = 6.23	<0.001*	η² = 0.032
	31-45	36.7 ± 9.1			
	46-60	34.2 ± 8.7			
	>60	31.8 ± 9.5			
Sex	Male	35.9 ± 8.9	t (563) = -1.68	0.094	d = 0.14
	Female	37.2 ± 9.3			
Educational status	No formal education	31.2 ± 8.4	F (4,560) = 14.86	<0.001*	η² = 0.096
	Primary school	34.8 ± 8.7			
	Secondary school	37.9 ± 8.5			
	Higher secondary	40.6 ± 8.3			
	Graduate and above	43.8 ± 7.9			
Occupation	Farmer	33.4 ± 9.1	F (6,558) = 4.17	<0.001*	η² = 0.043
	Daily wage worker	34.2 ± 9.3			
	Homemaker	36.8 ± 8.7			
	Government/private employee	41.2 ± 8.2			
	Business	39.7 ± 8.5			
	Student	38.9 ± 8.8			
	Unemployed	35.1 ± 9.6			
Monthly family income	<₹10,000	30.8 ± 8.2	F (4,560) = 22.43	<0.001*	η² = 0.138
	₹10,001-20,000	34.7 ± 8.6			
	₹20,001-40,000	39.2 ± 8.1			
	₹40,001-60,000	42.8 ± 7.8			
	>₹60,000	46.5 ± 7.3			

Domain-wise access scores and questionnaire reliability

The mean overall access score was 39.6 ± 10.8, indicating a moderate level of access to essential burn management medications. Among the five evaluated domains, accessibility demonstrated the highest mean score (8.8 ± 2.4), whereas affordability had the lowest (7.2 ± 2.8), suggesting that treatment cost remained the principal barrier to medication access. The questionnaire demonstrated excellent internal consistency, with an overall Cronbach's alpha of 0.88 (95% confidence interval {CI}: 0.86-0.90), while domain-specific Cronbach's alpha values ranged from 0.76 to 0.82 (Table 3). The test-retest reliability also demonstrated good reproducibility, with an ICC of 0.84 (95% CI: 0.78-0.89).

**Table 3:** Domain-wise scores and internal consistency of the questionnaire assessing access to essential medications for burn management. Data are presented as mean ± standard deviation (SD) or median (interquartile range {IQR}). Internal consistency was assessed using Cronbach's alpha with 95% confidence intervals (CI).

Domain	Number of items	Access score, mean ± SD	Median (IQR)	Cronbach's α	95% CI for α
Accessibility	3	8.8 ± 2.4	9.0 (7.0-11.0)	0.82	0.79-0.85
Affordability	3	7.2 ± 2.8	7.0 (5.0-9.0)	0.79	0.76-0.82
Availability	3	7.9 ± 2.6	8.0 (6.0-10.0)	0.81	0.78-0.84
Utilization	3	8.4 ± 2.3	8.0 (7.0-10.0)	0.76	0.72-0.80
Awareness	3	7.3 ± 2.5	7.0 (5.0-9.0)	0.78	0.74-0.82
Total access score	15	39.6 ± 10.8	40.0 (31.0-48.0)	0.88	0.86-0.90

Correlation analysis

The Pearson's correlation matrix is shown in Figure 1. Significant positive correlations were observed among all five access domains (p < 0.001). Accessibility showed the strongest correlation with the total access score (r = 0.82), followed by availability (r = 0.81) and affordability (r = 0.79). Monthly family income demonstrated the strongest positive correlation with overall access (r = 0.45), followed by educational status (r = 0.39). Conversely, age was negatively correlated with the total access score (r = -0.31), indicating reduced access among older participants.

**Figure 1:**
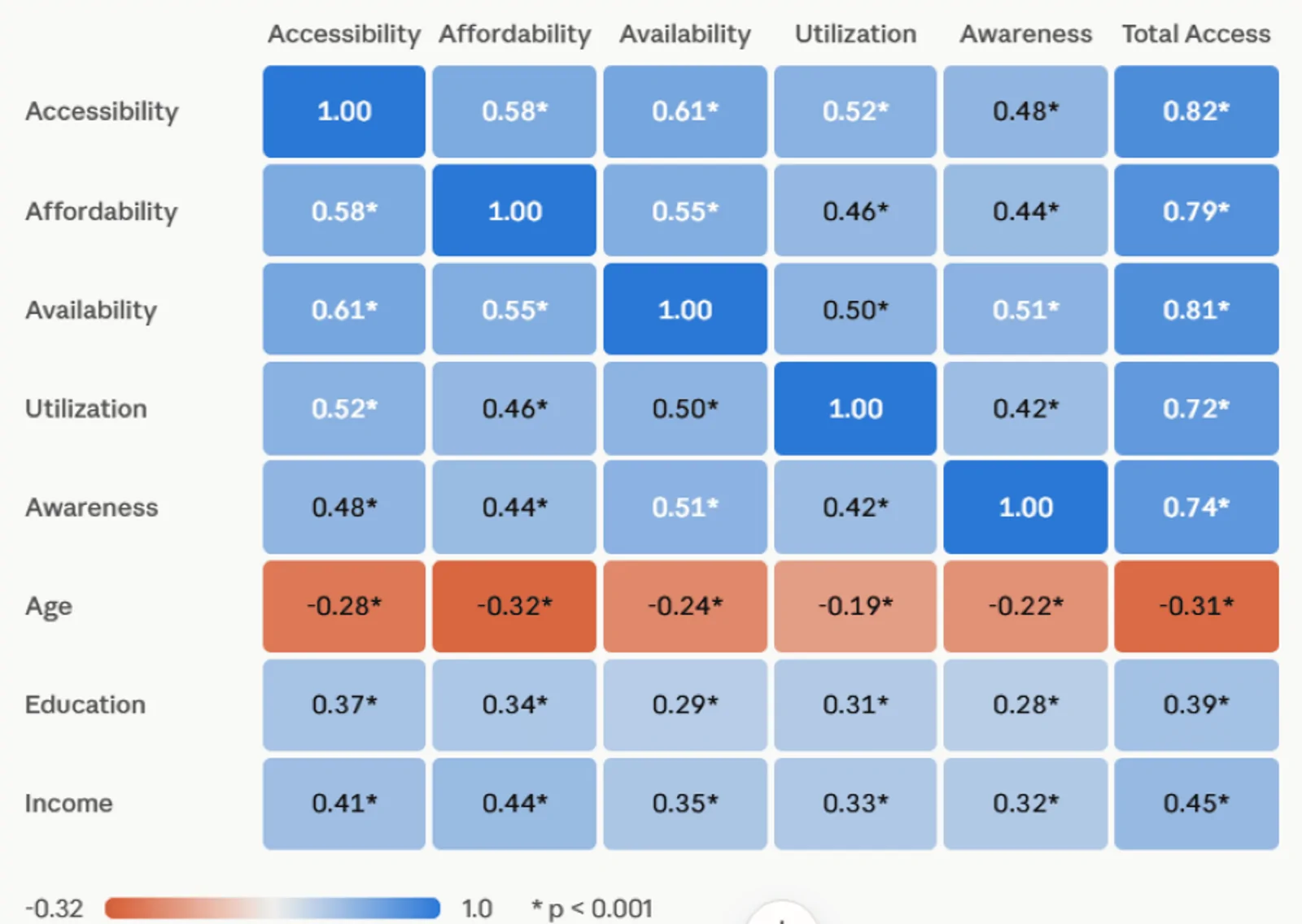
Correlation matrix of domain scores and demographic variables. Data presented as Pearson's correlation (r). *Statistical significance (p < 0.05).

Multiple linear regression analysis

The model was statistically significant (p < 0.001) and explained 41.2% of the variance in the total access scores (adjusted R² = 0.394). Higher educational attainment and higher monthly family income were the strongest independent predictors of better access. Graduates had significantly higher access scores than those without formal education (β = 11.34; p < 0.001). Similarly, respondents with a monthly family income of >₹60,000 demonstrated significantly better access than those earning <₹10,000 (β = 13.48; p < 0.001). Increasing age independently predicted poorer access, with participants aged >60 years exhibiting significantly lower access scores than those aged 18-30 years (β = -6.87; p < 0.001). Employment in the government or private sector was associated with significantly better access (β = 5.23; p = 0.002), whereas sex was not an independent predictor after adjustment (p = 0.154) (Table 4).

**Table 4:** Multiple linear regression analysis identifying predictors of access to essential medications for burn management (N = 565). The regression model was adjusted for age, sex, educational status, occupation, and monthly family income. *A two-tailed p-value of <0.05 was considered statistically significant. β, regression coefficient; CI, confidence interval; η², eta squared

Variable	Unstandardized β	Standard error	Standardized β	T-value	P-value	95% CI for β	Partial η²
Age (reference: 18-30)							
31-45	-2.18	1.36	-0.09	-1.6	0.11	-4.85 to 0.49	0.005
46-60	-4.52	1.48	-0.15	-3.05	0.002*	-7.42 to -1.62	0.016
>60	-6.87	1.72	-0.18	-3.99	<0.001*	-10.24 to -3.50	0.028
Gender (reference: male)							
Female	1.24	0.87	0.06	1.43	0.154	-0.47 to 2.95	0.004
Educational status (reference: no formal)							
Primary school	3.64	1.21	0.13	3.01	0.003*	1.27 to 6.01	0.016
Secondary school	6.21	1.28	0.2	4.85	<0.001*	3.70 to 8.72	0.04
Higher secondary	8.95	1.45	0.24	6.17	<0.001*	6.10 to 11.80	0.063
Graduate and above	11.34	1.67	0.27	6.79	<0.001*	8.06 to 14.62	0.076
Occupation (reference: farmer)							
Daily wage worker	0.87	1.32	0.03	0.66	0.51	-1.72 to 3.46	0.001
Homemaker	2.46	1.38	0.08	1.78	0.075	-0.25 to 5.17	0.006
Government/private employee	5.23	1.72	0.14	3.04	0.002*	1.85 to 8.61	0.016
Business	4.18	1.89	0.09	2.21	0.028*	0.47 to 7.89	0.009
Student	3.95	2.14	0.07	1.85	0.065	-0.25 to 8.15	0.006
Unemployed	1.78	2.21	0.03	0.81	0.419	-2.55 to 6.11	0.001
Monthly income (reference: <₹10,000)							
₹10,001-20,000	4.12	1.19	0.15	3.46	0.001*	1.78 to 6.46	0.021
₹20,001-40,000	7.85	1.34	0.26	5.86	<0.001*	5.22 to 10.48	0.058
₹40,001-60,000	10.62	1.63	0.26	6.52	<0.001*	7.42 to 13.82	0.07
>₹60,000	13.48	1.86	0.29	7.25	<0.001*	9.83 to 17.13	0.086

## Discussion

This community-based cross-sectional survey evaluated access to essential burn management medications among adults residing in rural communities and demonstrated an overall moderate level of access, with a mean access score of 39.6 ± 10.8. While the findings indicate that essential burn medications are available to a considerable proportion of the rural population, significant disparities remain across socioeconomic groups. Access was significantly influenced by age, educational status, occupation, and household income, whereas sex was not associated with access to medication. These findings highlight that socioeconomic and educational inequalities continue to play a major role in determining equitable access to burn care in rural areas.

Among the five evaluated domains, accessibility achieved the highest mean score, suggesting that primary healthcare facilities and local pharmacies have improved the geographical availability of essential burn medications in the region. This observation may reflect the expansion of primary healthcare services and government-supported medicine distribution programs in rural India over the past decade [7]. Nevertheless, geographical proximity alone does not ensure effective treatment, as medication access is also influenced by financial affordability, patient awareness, and the continuity of medicine supply. Similar observations have been reported in a previous community-based study, which showed that improvements in the physical availability of medicines do not necessarily eliminate barriers related to treatment completion and healthcare utilization [10].

A prospective observational study conducted across five Indian states reported that although emergency medical services transported 94.3% of critically burned patients to hospitals within two hours, the 30-day mortality remained high (64.5%), particularly among women with non-accidental burns. The study concluded that improving timely access to definitive burn care, strengthening critical care services, and implementing burn prevention strategies are essential for reducing burn-related mortality in India [11].

The affordability domain recorded the lowest mean score, indicating that treatment cost remains the principal obstacle to obtaining essential medications for burn management in India. Even when medicines are available at nearby health facilities, indirect expenses, including transportation costs, wage loss, and the need to purchase unavailable medications from private pharmacies, may discourage patients from seeking timely treatment or completing prescribed therapy [12]. Financial barriers are particularly relevant in rural populations, where household income is often limited and health expenditures are largely out of pocket. Therefore, the present findings emphasize that improving medicine availability alone may not sufficiently address inequities unless accompanied by strategies that reduce the economic burden of burn care [13].

Educational status was one of the strongest determinants of medication access. Participants with higher educational attainment had significantly better access scores than those with little or no formal education. Education likely improves health literacy, the awareness of available healthcare services, the understanding of prescribed treatments, and confidence in navigating healthcare systems. Individuals with higher educational attainment are also more likely to recognize the importance of early burn management and adhere to prescribed medications, thereby improving treatment outcomes [14].

Monthly household income was the strongest socioeconomic predictor of access to medication. Participants belonging to higher-income groups demonstrated substantially better access than those with lower incomes, and income remained an independent predictor, even after adjusting for other demographic variables. The present study identified affordability as the weakest domain of medication access, indicating that financial barriers remain a major obstacle to obtaining essential medications for burn treatment. Previous evidence suggests that reducing patients' out-of-pocket medication costs through financial assistance programs significantly improves medication adherence, persistence, and treatment continuation, emphasizing the importance of financial support mechanisms in achieving equitable access to essential medicines [15].

Age also significantly influenced medication access, with older adults demonstrating poorer access than younger adults. Several factors may explain this observation, including reduced mobility, transportation difficulties, dependence on caregivers, lower health literacy, and the presence of multiple chronic illnesses competing for limited financial resources. Therefore, older adults residing in rural communities may experience greater challenges in obtaining timely burn care and essential medications, highlighting the need for targeted outreach services and community-based healthcare support for older populations [16].

Interestingly, sex was not significantly associated with access to burn medications in either the univariate or multivariable analyses. This finding suggests that, within the surveyed communities, barriers to medication access are primarily driven by socioeconomic and educational factors rather than sex differences. Although sex disparities in healthcare utilization have been documented in some regions, the present findings indicate that access to burn medications may have become relatively equitable between men and women within the study setting [17].

The questionnaire demonstrated satisfactory content validity and good reliability, with an overall Cronbach's alpha of 0.88 and good test-retest reliability (ICC = 0.84). The high test-retest reliability further supports the stability and reproducibility of the instrument, indicating that it may serve as a useful, standardized tool for future community-based assessments of medication access in burn care. Reliable assessment instruments are essential for identifying healthcare gaps and monitoring the effectiveness of public health interventions. As evidence specifically addressing access to essential medications for burn management in rural communities is limited, the observed associations were interpreted in the context of available evidence on medication access, socioeconomic barriers, health literacy, and healthcare utilization, while the proposed explanations should be regarded as hypotheses rather than causal conclusions.

The present study has several potential implications for rural medication access. The finding that affordability was the lowest-scoring access domain highlights financial protection, strengthening medicine supply systems, and government-supported drug distribution as potential areas for policy consideration. However, the effectiveness of these approaches was not evaluated in the present study and therefore requires assessment in future intervention research. Community-based education focusing on burn first aid, timely healthcare-seeking, and appropriate medication use may also represent potential strategies for addressing gaps in awareness and medication use, particularly among individuals with lower educational attainment [18,19]. Further research could evaluate whether such interventions improve medication access and related care practices. In addition, older adults and economically disadvantaged populations may warrant particular attention in future community-based programs because these groups demonstrated poorer access scores in the present study.

This study had certain limitations. Its cross-sectional design precludes causal inferences between demographic factors and medication access. Information was based on participant self-report and may therefore be subject to recall and social desirability bias. Furthermore, the survey was conducted in rural communities surrounding a single institution, which may limit the generalizability of the findings to other geographical regions and urban populations. Participants were not required to have personal or household experience with burn injury or prior use of burn management medications. Therefore, responses to items concerning medication availability, affordability, and utilization may partly reflect perceived or reported community-level access rather than direct treatment experience. The access scores should consequently be interpreted as measures of perceived access within the rural population rather than as direct measures of individual treatment experience.

The study did not assess the availability of individual named burn medications or verify medication stocks at healthcare facilities; therefore, the findings reflect perceived access to burn management medications rather than the availability of specific medicines. Although the multistage cluster sampling design was accounted for during sample-size estimation using a design effect of 1.3, within-cluster correlation at the village/administrative-block level was not explicitly incorporated into the regression analysis, which may have resulted in underestimated standard errors and should be considered when interpreting the precision of the regression estimates. The access-score categories were based on the equal-interval classification of the possible score range and have not been externally validated; therefore, these categories should be interpreted as descriptive rather than definitive measures of the adequacy of medication access. Finally, this study evaluated perceived access rather than objectively measuring medicine availability within healthcare facilities.

Future research should include multicenter studies involving diverse geographical regions and longitudinal designs to evaluate changes in medication access over time. Incorporating healthcare facility audits, pharmacy inventory assessments, and qualitative interviews with patients and healthcare providers would provide a more comprehensive understanding of the barriers to accessing burn medication. Evaluating the effectiveness of government medicine distribution programs and community-based educational interventions may help identify sustainable strategies for improving equitable access to burn care in underserved populations.

## Conclusions

This study demonstrated a moderate level of access to essential medications for burn management among rural adults, with affordability remaining the greatest barrier, despite relatively good geographical accessibility. Educational attainment, household income, occupation, and age were significantly associated with medication access, whereas sex had no independent effect. These findings highlight the need for policies to improve the affordability of medicines, strengthen rural healthcare delivery, and enhance community awareness. Addressing socioeconomic disparities through targeted public health interventions may substantially improve equitable access to burn medications and ultimately contribute to better burn-care outcomes.

## Appendices

Table 5 shows the questionnaire used in the study.

**Table 5:** Questionnaire used in the study. Credit: Vidya Kiran Bammidi, K Murali, J. Dhanuja Rani, Parth Suthar, and Rahul VC Tiwari.

Part 1: Demographic	
Q1. Age	□ 18-30 years
	□ 31-45 years
	□ 46-60 years
	□ >60 years
Q2. Gender	□ Male
	□ Female
Q3. Educational status	□ No formal education
	□ Primary school
	□ Secondary school
	□ Higher secondary
	□ Graduate and above
Q4. Occupation	□ Farmer
	□ Daily wage worker
	□ Homemaker
	□ Government/private employee
	□ Business
	□ Student
	□ Unemployed
Q5. Monthly family income	□ <₹10,000
	□ ₹10,001-20,000
	□ ₹20,001-40,000
	□ ₹40,001-60,000
	□ >₹60,000
Part 2: Access to essential medications for burn management	
Accessibility domain	
1. Essential medicines required for treating burns are available at the nearest healthcare facility.	□ Never □ Rarely □ Sometimes □ Often □ Always
2. Burn medicines are available whenever they are needed.	□ Never □ Rarely □ Sometimes □ Often □ Always
3. I can obtain burn medicines without travelling long distances.	□ Never □ Rarely □ Sometimes □ Often □ Always
Affordability domain	
4. I can afford the medicines required for burn treatment.	□ Never □ Rarely □ Sometimes □ Often □ Always
5. The cost of burn medicines prevents me from completing treatment (reverse-scored).	□ Never □ Rarely □ Sometimes □ Often □ Always
6. Government hospitals provide burn medicines free of cost whenever required.	□ Never □ Rarely □ Sometimes □ Often □ Always
Availability domain	
7. Local pharmacies usually stock medicines required for burn treatment.	□ Never □ Rarely □ Sometimes □ Often □ Always
8. Health centers maintain an adequate supply of burn medicines.	□ Never □ Rarely □ Sometimes □ Often □ Always
9. I receive all prescribed burn medicines without stock shortages.	□ Never □ Rarely □ Sometimes □ Often □ Always
Utilization domain	
10. I complete the prescribed course of burn medicines.	□ Never □ Rarely □ Sometimes □ Often □ Always
11. I seek medical care promptly after a burn injury.	□ Never □ Rarely □ Sometimes □ Often □ Always
12. Healthcare providers clearly explain how to use burn medicines.	□ Never □ Rarely □ Sometimes □ Often □ Always
Awareness domain	
13. I know which medicines should be used for burn treatment.	□ Never □ Rarely □ Sometimes □ Often □ Always
14. I know where essential burn medicines can be obtained in my area.	□ Never □ Rarely □ Sometimes □ Often □ Always
15. I trust the medicines provided by government healthcare facilities for burn treatment.	□ Never □ Rarely □ Sometimes □ Often □ Always
